# Variation in Thermal Sensitivity and Thermal Tolerances in an Invasive Species across a Climatic Gradient: Lessons from the Land Snail *Cornu aspersum*

**DOI:** 10.1371/journal.pone.0070662

**Published:** 2013-08-05

**Authors:** Juan Diego Gaitán-Espitia, María Belén Arias, Marco A. Lardies, Roberto F. Nespolo

**Affiliations:** 1 Instituto de Ciencias Ambientales y Evolutivas, Universidad Austral de Chile, Valdivia, Chile; 2 Programa de Doctorado en Ciencias mención Ecología y Evolución, Facultad de Ciencias, Universidad Austral de Chile, Valdivia, Chile; 3 Departamento de Ciencias, Facultad de Artes Liberales, Universidad Adolfo Ibañez, Peñalolen, Santiago, Chile; 4 Departamento de Ciencias, Facultad de Ingeniería y Ciencias, Universidad Adolfo Ibañez, Peñalolen, Santiago, Chile; University of Missouri, United States of America

## Abstract

The ability of organisms to perform at different temperatures could be described by a continuous nonlinear reaction norm (i.e., thermal performance curve, TPC), in which the phenotypic trait value varies as a function of temperature. Almost any shift in the parameters of this performance curve could highlight the direct effect of temperature on organism fitness, providing a powerful framework for testing thermal adaptation hypotheses. Inter-and intraspecific differences in this performance curve are also reflected in thermal tolerances limits (e.g., critical and lethal limits), influencing the biogeographic patterns of species’ distribution. Within this context, here we investigated the intraspecific variation in thermal sensitivities and thermal tolerances in three populations of the invasive snail *Cornu aspersum* across a geographical gradient, characterized by different climatic conditions. Thus, we examined population differentiation in the TPCs, thermal-coma recovery times, expression of heat-shock proteins and standard metabolic rate (i.e., energetic costs of physiological differentiation). We tested two competing hypotheses regarding thermal adaptation (the “hotter is better” and the generalist-specialist trade-offs). Our results show that the differences in thermal sensitivity among populations of *C. aspersum* follow a latitudinal pattern, which is likely the result of a combination of thermodynamic constraints (“hotter is better”) and thermal adaptations to their local environments (generalist-specialist trade-offs). This finding is also consistent with some thermal tolerance indices such as the Heat-Shock Protein Response and the recovery time from chill-coma. However, mixed responses in the evaluated traits suggest that thermal adaptation in this species is not complete, as we were not able to detect any differences in neither energetic costs of physiological differentiation among populations, nor in the heat-coma recovery.

## Introduction

Temperature is an important environmental factor that affects species distribution [1], [2], influencing at the same time all life functions of organisms through changes in the rates of physiological and biochemical processes [3]–[5]. Generally, the thermal sensitivity of most biological rate processes at the whole-organism level (i.e., rates of locomotion, growth, development, and fitness) operates within the ranges of critical temperature extremes, with the performance of a biological trait gradually increasing with temperature from a critical minimum (CT_min_) to an optimum (T_opt_) before dropping precipitously as temperature approaches a critical maximum (CT_max_) [6], [7]. Moreover, it is well known that the ability of an organism to perform at different temperatures (i.e., their thermal sensitivity) can be described by a continuous nonlinear reaction pattern (i.e., thermal performance curve, TPC) in which the phenotypic trait value varies as a function of temperature [8]–[10]. These thermal reaction patterns characterize the direct effects of temperature on performance [11], providing a powerful framework for testing thermal adaptation hypotheses [12].

A TPC is typically described in terms of three key parameters: (1) the optimal temperature (or thermal optimum), which defines the temperature that optimizes performance; (2) the thermal breadth (or performance breadth), which defines the range of temperatures that permits some level of performance; and (3) the maximal performance, which defines the level of performance at the optimal temperature [13]. Variation in these parameters among species, populations or genotypes can be used to describe the variation of their thermal sensitivities [10], [14], [15]. In principle, adaptive evolution or phenotypic plasticity can modify a TPC by means of vertical shifts in the shape that produces changes in average performance or fitness, by horizontal shifts that produce changes in the T_opt_, or by width shifts that produce changes in the niche width [9], [15]. In keeping with this line of thinking, it is believed that these kinds of changes have an effect on fitness or performance trade-offs [9], [12] that impose a cost to thermal specialization and result in either specialists that are well adapted to local conditions but poorly adapted to alternative environments, or generalists that are broadly adapted to a range of environments but not particularly well adapted to any particular one (i.e., generalist-specialist trade-off) [9], [16], [17]. This is particularly interesting in invasive species, since they exhibit great physiological tolerance to thermal variation [18], [19], as well as rapid phenotypic responses and adaptation to novel environments [20], [21].

Generally, invasive species have shown rapid niche shifts that could be reflected as width shifts in their TPCs [22] that are mainly associated with the generalist-specialist mode of thermal evolution [7]. However, thermal adaptation exhibited by invasive species to novel environments is a much more complex process that involves thermodynamic constrains [23], due to the effects of temperature on the kinetic energy of molecules, yielding faster biochemical reactions at higher temperatures than at lower ones [3], [24]. Accordingly, some authors have argued that populations which are thermally adapted to warmer environments will perform better than those populations adapted to colder environments (e.g., higher latitudes), leading to the notion that “hotter is better” [1], [9], [13], [23], [25]–[27]. As a result of this thermodynamic reasoning, the maximum potential performance or fitness of adapted organisms will be greater in a warmer environment than in a cooler one [7].

In addition to thermal sensitivities, thermal tolerance could be an important constraint in thermal niche expansions. In fact, chronic exposure to extreme temperatures induces thermal stress, seen as a deleterious effect on organismal performance [28]. However, many ectothermic invasive species display phenotypic plasticity to compensate drastic changes in environmental conditions [29]. Such plastic responses could be viewed on a short-term scale, as reversible changes within an individual (i.e., phenotypic flexibility), or on a long-term scale, as irreversible changes that result from developmental processes (i.e., developmental plasticity) [30], [31]. For instance, it is well known that ectotherms effectively compensate variation in their body temperatures using various behavioral adjustments [32], or by modification of their molecular and cellular structures to maintain performance as their environments change [3], [33]. One of the main molecular responses that is activated in a cell under thermal stress is the heat shock protein response (HSPR), a genetic activation that occurs at the cell level in response to abnormal, stressfully high or low temperatures [34], [35]. As molecular chaperones, HSPs stabilize denaturing proteins and refold those that have already been denatured [36], thus, the HSPR could be considered an ecologically and evolutionarily important factor in thermal adaptation, setting thermal tolerance limits and improving an animal’s tolerance to thermal stress [36]–[39].

**Figure 1:**
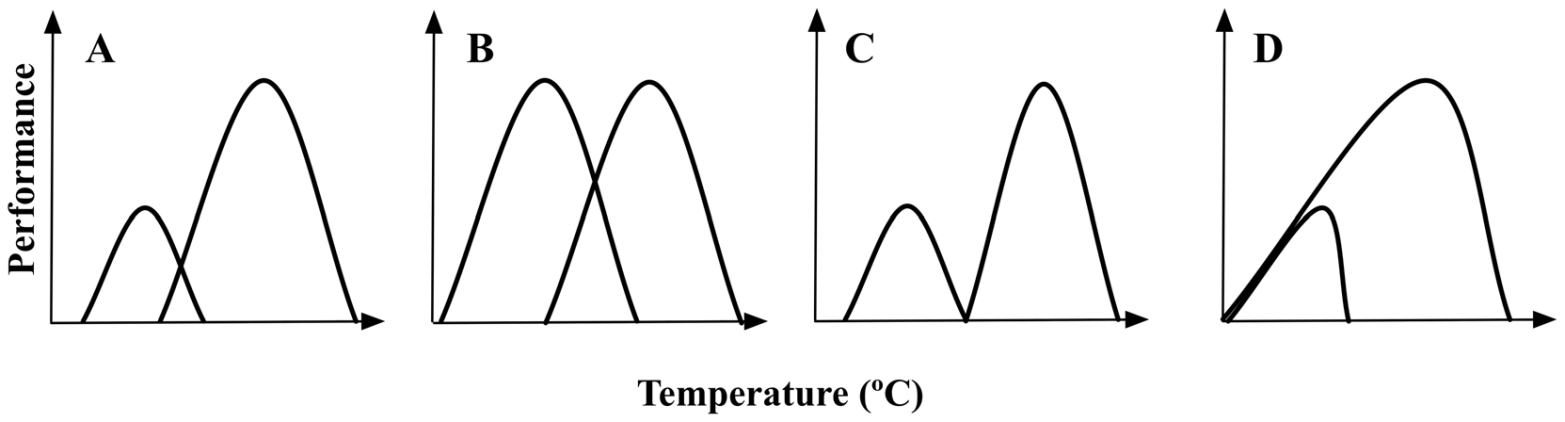
Hypotheses related to variation in the thermal sensitivity in natural populations. A) The hotter is better hypothesis predicts that populations adapted to warmer temperatures will show increased maximum performance compared to those adapted to colder temperatures. B) If thermodynamic constraints do not limit adaptation to temperature, then adaptation to warmer and colder environments is predicted to result in equal maximal performance. C) In combination with hotter is better, generalist-specialist trade-offs predict that populations adapted to warmer temperatures will have a narrow temperature range. D) If generalist-specialist trade-offs or other environmental effects do not constrain temperature adaptation, then populations adapted to the warmer temperatures are predicted to have the wider temperature ranges. Modified from [26].

In this work we study the population differentiation in thermal performance of the invasive land snail *Cornu aspersum*, which is originally from warm environments in Mediterranean countries [40], [41]. Specifically, we examined the intraspecific thermal sensitivity and thermal tolerance variation in populations of *C. aspersum* across a climatic/geographic gradient along Chile, by mean of the analysis of the TPCs, thermal-coma recovery times, expression of heat-shock proteins and standard metabolic rate (i.e., energetic costs of physiological differentiation). We tested two competing hypotheses regarding thermal adaptation. The “hotter is better” hypothesis predicts that populations of *C. aspersum* living at low latitudes and experiencing warmer temperatures will show higher maximum performance than those living at high latitudes and experiencing colder temperatures (Fig. 1A). If thermodynamic constraints do not limit adaptation to temperature, then populations in warmer and colder environments are expected to have equal maximum performance (Fig. 1B). As with hotter is better, the generalist-specialist trade-off hypothesis predicts that populations adapted to warmer temperatures will have a narrower temperature range (Fig. 1C). If generalist-specialist trade-offs or other environmental effects do not constrain temperature adaptation, then populations of *C. aspersum* adapted to warmer temperatures are expected to have wider temperature ranges (Fig. 1D) [26], [27].

## Materials and Methods

### Study Organism

The common garden snail *Cornu aspersum* is originally from Mediterranean countries [40], and represents a typical anthropochorous form (i.e., species with of human-mediated dispersion) with widespread world distribution, in many regions having Mediterranean, temperate or even subtropical climates [42]. This land snail exhibits a great shell variation across geographical regions [43] and is characterized by physiological and morphological adaptations (e.g., metabolic depression, reduction of water-loss rate, thicker shell, reduced shell aperture, thicker epiphragm and supercooling ability) that enable it to inhabit dryer, colder and/or hotter environments by increasing the amount of time the snail can remain inactive [44]–[46]. Additionally, empirical evidence suggests that *C. aspersum* snails are energetically constrained by the mode of locomotion [47] and the cost of shell production during ontogeny [48].

### Populations and Climatic Data

**Figure 2:**
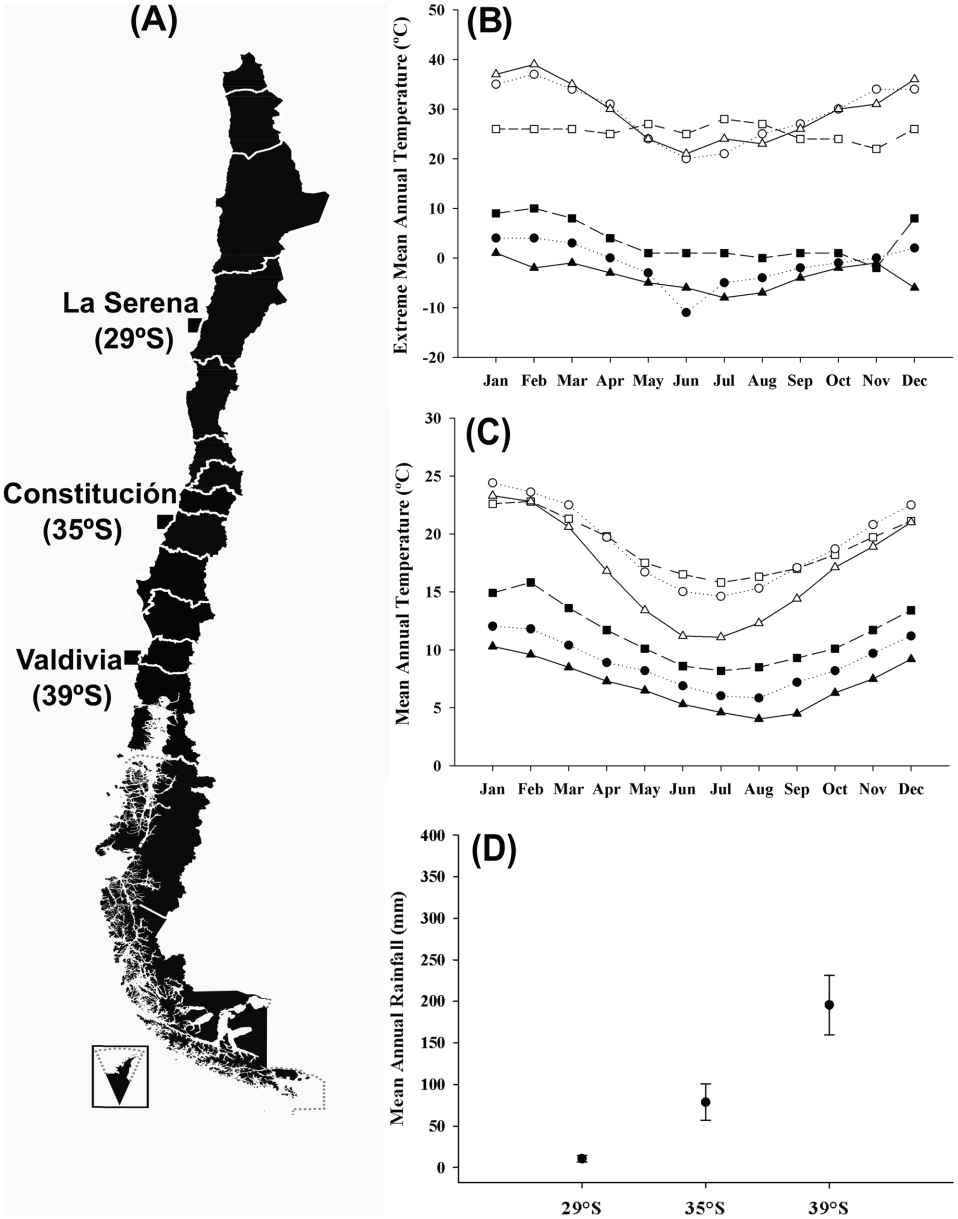
Populations and climatic characteristics in the geographic gradient. Map of Chile showing the locations of the three *C. aspersum* populations (A). Extreme mean temperatures for month (B), and overall mean monthly temperatures (C) for La Serena (Square), Constitución (Circle) and Valdivia (Triangle). Filled symbols are the minimum values whereas open symbols are the maximum values. Latitudinal distribution of the mean annual rainfall (D). Error bars represent one standard error.

Three coastal populations of the land snail *C. aspersum* were selected from a latitudinal gradient within a range of approximately 1300 Km from northern to southern Chile: La Serena (29°54′ S, 71°15′ W), Constitución (35°20′ S, 72°25′ W) and Valdivia (39° 38′ S, 73° 5′ W) (Fig. 2A). We selected these localities based on their climatic characteristics and differences in thermal variability (Fig. 2C–2D). La Serena is considered a mesomediterranean arid environment [49], characterized by a mean annual temperature of 13.43±2.17°C. Constitución is a mesomediterranean subhumid environment [49], with a mean annual temperature of 12.36±2.65°C, whereas Valdivia is considered a mesotemperate perhumid environment [49], with a mean annual temperature of 10.79±3.05°C. Climatic data were downloaded from http://www.meteochile.gob.cl.

### Animal Maintenance and Husbandry

Six hundred adult snails of approximately same body mass (mean ± SD; 4.21±0.63 g, with an approximate age of less than one year) [48] were collected by hand from gardens and parks in each one of the three localities (Total snails = 1800), placed in plastic containers and transferred to the laboratory at the Universidad Austral de Chile in Valdivia. According to their original populations, animals were identified with shell numbers and housed in plastic cages (60×60×13 cm) filled with 10 cm of humid soil. Snails were maintained at densities of 50 animals per cage and were fed *ad libitum* with a mix of corn/wheat flour and calcium carbonate (1∶1:0.3). The temperature and photoperiod for rearing conditions were 20°C and 16∶8 L:D, respectively. Relative humidity was maintained at high levels by sprinkling the interior of the boxes with water every day. As a mode of separating environmental effects from local differentiation, we used a common garden experiment with homogeneous laboratory acclimation of the three populations, during three months. Hence, any observed phenotypic difference will be attributable to the source populations [50], [51].

### Respirometry and Water Balance

A total of 314 adult snails (103 from La Serena, 108 from Constitución and 103 from Valdivia) were randomly selected for metabolic measurements. In this study, standard metabolic rate (SMR), the obligatory maintenance energy cost in ectotherms [52], was measured as the rate of carbon dioxide production using an open system, as described by Gaitán-Espitia et al (2012) [53]. Briefly, CO_2_ production at 20°C was measured continuously with an infrared CO_2_ analyzer (LI-COR 7000 Sable System®) capable of resolving differences of up to 1 part per million of CO_2_ in air, connected to a computerized data-acquisition system (Expe Data software, Sable Systems), similar to one used by [54]. The analyzer was calibrated periodically against two kinds of gases (CO_2_-free air and a commercial mix of 291 PPM of CO_2_). A Sable System eight-channel multiplexer was used for obtaining the measurements, 5 chambers with individual snails and 3 chambers for baseline measurements (before and after all recordings), which allowed corrections for possible drift (although it was almost non-existent between baselines). The arrangement of the respirometry system was as follows: ambient air was first pumped at 100 ml min^−1^ through a Drierite/soda lime column, to remove water vapor and CO_2_. The air was then passed through a single flow-meter maintaining a constant (±1%) flow rate through the respirometry chambers. CO_2_-free air was kept flowing at all times through all chambers, while each was being measured. We used transparent metabolic chambers (60 ml), each one with a 100% hydrated snail. Animal activity was visually monitored at intervals of ca. 10 min during measurements, which had a total duration of 45 minutes each. Activity was rarely observed during respirometry measurements, and the data of active animals were not included in the analysis. Records were automatically transformed by a macro program recorded in the ExpeData software (Sable Systems), to transform the measure from part per-million to ml-CO2 per hour. Metabolic rates were analyzed using the formula [55]:
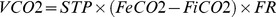



Where: FeCO2 is the excurrent fractional concentration of CO2, FiCO2 is the input fractional concentration of CO2, FR is the flow rate in ml min-1, STP is the correction factor for standard conditions of temperature and pressure (which for mass flowmeters is equal to 1). A minor correction term in the equation, which includes respiratory quotient (RQ), was omitted because its effect is are less than 1% with RQ values above 0.85 [55].

We eliminated the first 10 minutes of the recordings (600 samples) to avoid any noise or erroneous recordings generated by animal manipulation. From each individual recording, we extracted three variables: complete average of each transformed recording (VCO_2__Mean_), which is used here as a measure of SMR; the average of the one-minute steady state of minimum VCO_2_ production (VCO_2__min_); and the average of the one-minute steady state of maximum VCO_2_ production (VCO_2__max_). In order to achieve a post-absorptive state, metabolic rate was measured in 18 h food deprived individuals [29]. All metabolic trials were performed during the day, when land snails are inactive, corresponding to the rest phase in this species [48]. All individuals were weighed at the beginning and at the end of the test period (i.e., 45 min), recording the mean body mass (Mb) and body size. Additionally, to determine the extent of the ability to maintain water balance (i.e., body dehydration, BD), the snails were weighed at the beginning of each trial, when the snails were fully hydrated, and at the end of the test period (i.e., 45 min), recording the mean Mb and noting any differences between initial and final Mb as a measure of body water loss.

### Experimental Procedures

After the respirometric trials, snails were randomly separated into two experimental groups. In the first group, 930 adult snails (310 from each population) were assigned to thermal sensitivity analysis and estimation of the TPCs. In the second group, 145 adult snails (n = 50 La Serena, n = 48 Constitución and n = 47 Valdivia) were selected to explore the thermal tolerance limits (i.e., heat- and cold-shock responses) of the snail populations. Because locomotion is one of the most important components in the gastropod’s energy budget [47], [56] and is considered to be a measure of the relationship between organismal performance and environmental temperature [57], we used rollover speed (i.e. the time taken for an individual to change from an inverse to an upright position) as a measure of the locomotor performance capacity [58], [59] in the snails. Additionally, this trait has been described as one of the main gastropod behaviors used to avoid and escape predators, as well as in stressful situations by means of instantaneous reactions that involve shell twisting or ﬂeeing movements [60].

### Thermal Sensitivity and Performance

In group 1, rollover speed was measured between −2°C and 42°C. In extreme temperatures of the thermal treatment (i.e., −2° to 7°C and 35° to 42°C), rollover speed was measured every 1°C, whereas at intermediate temperatures (i.e., 7°C to 35°C) it was measured every 2°C. At each experimental temperature (−2°C to 42°C), 10 snails per population group were assigned to a plastic chamber with 10 subdivisions (i.e., one subdivision for each single snail), placed in an incubator and maintained at the corresponding experimental temperature using a thermal bath (Labtech LCB-R20 precision ±0.1°C). Duration of thermal treatments was always 60 minutes of exposure. In the last 15 minutes, snails were turned over and if an individual was not in a thermal coma, it responded by returning to an upright position. Each snail was used for only one experimental temperature, to estimate the shape of the thermal performance curve for each population. Animals that died during this procedure were excluded from the experiment. At extreme temperatures, some individuals had zero performance. Those snails that exhibited a complete loss of righting response under extreme temperatures were considered to be in a thermal coma [58], [61]. Before measuring, individual body mass was recorded with an analytical balance (RADWAG MXA-5I1, with precision ±0.01 mg).

The mean rollover speed for each experimental temperature was calculated, with the aim of estimating thermal performance curves for each population. We used the TableCurve2D curve-fitting software (version 5.01; Systat Software, Inc.) for model fitting. We used unlimited number of terms and extracted µ_max_ (i.e., maximum rollover speed) and T_opt_ (i.e., optimum temperature) for each population (maximum of 100 iterations). We compared the fit of several functions (Table 1) that could describe snail performance as a function of temperature using the Akaike Information Criterion [14]. The AIC represents a balance between the likelihood explained by the model and the number of model parameters, with the best model minimizing AIC [62]. The ecophysiological characteristics of Critical thermal maximum (CT_Max_) and minimum (CT_Min_) were derived numerically as the intersection points of the resulting thermal performance curve with the temperature axis (μ = 0). Temperature breadth (T_br_) for each population was calculated with the mean values of rollover speed for each experimental temperature using the following equation [63]:
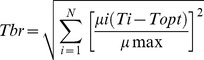
where N equals the number of temperatures and µ_i_ is the mean rollover speed at temperature Ti. Three indices of the thermal performance curves (i.e., maximum rollover speed, optimum temperature and breadth temperature) were used to compare the relationship of temperature and locomotor performance among populations in the latitudinal gradient.

**Table 1:** Comparison of functions used to describe the thermal performance curves of the three populations of the land snail *Cornu aspersum* across the latitudinal gradient, using Akaike’s information criterion (AIC).

Population	Function	K	AIC	λ_i_	w_i_	r^2^
La Serena	**Half-Gauss-Mod**	5	131.99	0	0.39	0.92
	Gaussian	4	132.50	0.51	0.30	0.91
	Weibull	5	132.67	0.68	0.27	0.92
	Exp-Mod Gaussian	5	136.99	5.01	0.03	0.93
	Lorentzian	4	142.05	10.06	0.00	0.94
Constitución						
	**Weibull**	5	168.56	0	0.62	0.96
	Gaussian	4	170.65	2.09	0.22	0.96
	Half-Gauss-Mod	5	171.42	2.86	0.15	0.96
	Exp-Mod Gaussian	4	177.21	8.65	0.01	0.97
	Lorentzian	5	192.48	23.92	0.00	0.98
Valdivia						
	**Gaussian**	4	161.26	0	0.88	0.95
	Weibull	5	166.64	5.37	0.06	0.96
	Half-Gauss-Mod	5	167.58	6.31	0.04	0.96
	Lorentzian	4	169.13	7.86	0.02	0.96

The function with the lowest AIC is the one that best describes the data. K = number of parameters in the function, λ_i_ = difference between a given model’s AIC and the lowest AIC, w_i_ = Akaike weight. Models in boldface were selected for analyses.

### Thermal Tolerance Limits

In order to examine heat- and cold-shock responses (i.e., recovery time from chill- and heat-coma) of snail populations in the climatic gradient, adult snails from group 2 were exposed to fixed experimental temperatures. Each snail was individually kept in a plastic chamber, placed inside an incubator and maintained at the corresponding experimental temperature using a thermal bath (Labtech LCB-R20 precision ±0.1°C). Duration of cold and heat treatments was similar to the previous procedure of thermal sensitivity (i.e., 60 minutes of exposure and an evaluation in the last 15 minutes). If animals did not show coordinated movements after 15 min, and were still inverted over their shells, they were considered in to be in a thermal coma [35], [64]. Temperature treatments were changed by 1°C until the snail was in a thermal-coma. Cold treatment was decreased from 7°C, whereas heat treatment was increased from 35°C. Only snails in thermal comas (i.e. unable to rollover or show coordinated movements) were brought to room temperature (20±2°C) and the time taken for each individual to recover its capacity to rollover or show coordinated movements was registered and referred to as “recovery time” [33], [35], [58]. To maximize our ability to detect and record recovery time, no more than six snails were assessed at a time. All animals that died or did not exhibit movements in the time established for recovery analysis were excluded from this study. Before any measurements were taken, the body mass of each snail was recorded with the aid of an analytical balance. For further analysis of Hsp70 protein expression, snails that showed heat-coma were dissected three to four hours after, and foot tissue was stored at –80°C (ULT Freezer Thermo model 702).

### Heat Shock Protein (HSP70) Expression

Due to the great effect of high experimental temperatures on snails’ survival, only those snails that recovered from the heat-coma were considered for stress protein analysis. Analysis of heat shock protein (HSP70) was developed following [65]. Samples were homogenized on ice in 500 mL of extraction buffer (50 mM Tris-HCL, pH 8.0, 150 mM NaCl, 1 mM PTC), triturated with an Ultra-Turrax tissue homogenizer, and centrifuged at 9500 g for 10 minutes at 4°C (BOECO Germany U-320 R). The total protein concentration in each supernatant was determined using Bio-Rad® Protein Assay (Method of Bradford) according to manufacturer’s instructions. Proteins were separated electrophoretically (Tetra cell Bio-Rad®) and analyzed by a SDS-PAGE mini gel (12% acrylamide- bisacrylamide) for 15 minutes at 80 V and 100 minutes at 120 V. Each line was loaded with 30 µg of total protein, treated previously with a denaturizing buffer at 37°C for 20 minutes in a water bath. Protein was transferred to pure 0.2 µm nitrocellulose membranes (Bio-RAD®), and the filter was blocked for one hour in a blocking buffer (0.05 M Tris-HCl, 0.15 M NaCl, 5% [w/v] non-fat milk) at room temperature. Membranes were soaked for 75 minutes at 350 mA in transfer buffer (Glycine 20%, Methanol). After blocking, the filters were washed in TBS for 5 minutes and incubated in the first antibody solution (mouse anti-human HSP70 Clone 3A3, Dianova®, Hamburg, Germany, dilution 1∶5000 in 10% horse serum in TBS) overnight at room temperature on a laboratory shaker. To verify that equal amounts of proteins were loaded onto the stacking gel, β-actin expression was estimated in each organism by Western blot using beta Actin Antibody (mAb, Mouse, GenScript®). Subsequently, nitrocellulose membranes were washed again in TBS for 5 minutes and incubated in a second antibody solution (pre-diluted Alkaline-Phoshatase conjugated Anti-Mouse, Invitrogen®) on the laboratory shaker for 2 hours. After repeated washings for 5 minutes in TBS, the antibody cross reaction was detected by staining with NBT/BCIP Reagent Kit (Invitrogen®) for 10 minutes at room temperature, and dried for 2 hours. Each gel contained a pre-stained protein ladder (Benchmark, GibcoGRL, Life Technologies, Gaithersburg, MD, USA) used as an electrophoresis control. Membranes were digitalized and analyzed with ImageJ software (NIH, Bethesda, Maryland, USA). To quantify the band intensities of Hsp70 from snail foot tissue, we used the relative expression of Hsp70/β-actin protein.

### Statistical Analysis

Prior to analyses, we tested for normality and homoscedasticity for all variables using the Lilliefors and Levene tests, respectively. VCO_2__Mean_, VCO_2__Max_ and BD were log10 transformed, whereas VCO_2__Min_ and Mb were square-root transformed before the analysis. A Pearson’s correlation test was conducted to evaluate potential correlation between body mass (Mb), metabolism (i.e., VCO_2Mean_, VCO_2max_ and VCO_2min_) and ability to maintain water balance (i.e., Body dehydration, BD) in *C. aspersum* across populations in the geographical gradient. Comparisons of M_b_, recovery time and HSP70 expression between populations were done using analysis of variance (ANOVA), whereas SMR and BD were analyzed by ANCOVA (mean M_b_ as covariate). Lastly, comparisons of locomotion performance (rollover speed) among the three populations were analyzed using a two-way ANOVA, with experimental temperature and populations as categorical predictors (Mb was not correlated to performance). Thermal performance curves were analyzed by (i) correlation test between thermal indices (i.e., µ_max_, T_opt_, T_br_, CT_min_ and CT_max_) and annual temperatures (i.e., mean and extreme minimum and maximum temperatures) for each population across the latitudinal gradient to explore thermal adaptation to their respective local environments; (ii) one-way ANOVA tests to compare µ_max_, T_opt_ and CTs of performance curves among populations. When differences in the means were significant at the P<0.05 level, they were also tested with a posteriori Tukey’s test (HSD). Finally, a Chi^2^ test was used to identify differences among populations in the temperatures at which snails were in thermal-coma. All analyses were run in the Statistica v7.0 software (StatSoft).

### Ethics Statement

This study did not involve endangered or protected species and was carried out in strict accordance with the recommendations in the Guide for the Care and Use of Laboratory Animals of the Comisión Nacional de Investigación Científica y Tecnológica de Chile (CONICYT). All experiments were conducted according to current Chilean law. The protocol was approved by the Committee on the Ethics of Animal Experiments of the Universidad Austral de Chile (Permit Number: 02-2011). Because snails were obtained from public parks and gardens, no specific permissions were required for any of the three locations involved in this study (La Serena, Constitución and Valdivia).

## Results

### Thermal Sensitivity and Thermal Tolerances

**Figure 3:**
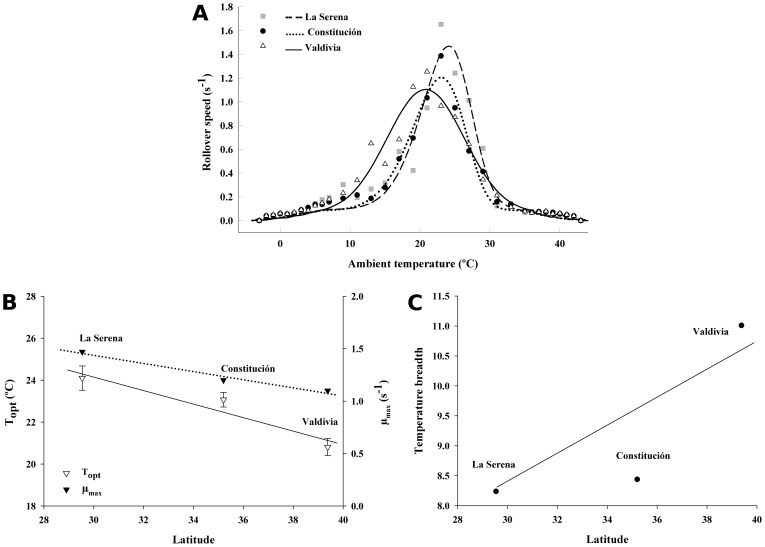
Geographic variation in thermal sensitivity of *C. aspersum*. A) Thermal performance curves for the three populations of the land snail *C. aspersum*. Latitudinal variation in the temperature optimum (B), and in the thermal breadth (C) of the land snail *C. aspersum*. Comparison of µ_max_ showed significant differences among populations (ANOVA, P<0.001). Similarly, the analysis of thermal optimum showed differences among populations (ANOVA, F_2,27_ = 9.14; P<0.01) with higher T_opt_ in snails from the lowest-latitude population and lower T_opt_ in snails from highest latitude population (Tukey’s HSD tests, P<0.001).

According to AIC, the Half-Gauss-Mod function was the best model explaining locomotor performance of snails from La Serena, whereas the Weibull and Gaussian functions were selected for Constitución and Valdivia, respectively (Table 1). The upper and lower bounds for critical temperatures were not correlated with annual mean or extreme temperatures in the latitudinal gradient. Differences among populations in these critical bounds (Table 2) were not statistically significant (One-Way ANOVA, CT_min_: F_2,27_ = 0.81, P = 0.46; CT_max_: F_2,27_ = 0.69, P = 0.51). Analysis of the locomotor performance between populations and experimental temperatures showed a significant interaction between both variables (Two-Way ANOVA, F_60,837_ = 1.95; P<0.001). Additionally, snails from Valdivia showed differences in rollover speed at 13, 19, 20 and 23°C compared to the other populations (Fig. 3A; Tukey’s HSD tests, P<0.001). Snails from La Serena showed differences at 25 and 27°C (Fig. 3A; Tukey’s HSD tests, P<0.001).

**Table 2:** Physiological variables measured in three populations of the land snail *C. aspersum* across a latitudinal gradient.

	La Serena	Constitución	Valdivia
**Latitude**	29°54′ S, 71°15′ W	35°20′ S, 72°25′ W	39° 38′ S, 73° 5′ W
**Body size (cm)**	25.25±0.34	25.69±0.39	26.03±0.38
**Body mass (g)**	4.53±0.15	5.08±0.19	5.33±0.20
**SMR (mL CO_2_ h^−1^)**	0.26±0.01	0.28±0.01	0.31±0.02
**SMR (µW s^−1^)**	379.91±20.5	359.36±18.4	374.9±25.43
**Body dehydration (%)**	3.58±0.39	3.44±0.40	2.18±0.21
**CT_min_ (**°**C)**	−2.63±0.18	−2.59±0.23	−2.77±0.15
**CT_max_ (**°**C)**	42.35±0.34	42.77±0.29	42.71±0.46
**T_opt_ (**°**C)**	24.10±0.58	23.07±0.35	20.81±0.41
**µ_max_**	1.47±0.06	1.20±0.03	1.10±0.04
**T_br_**	8.24	8.44	11.01

(Mean ± S.E.M).

Overall, the parameters of performance curves such as T_br_, T_opt_ and µ_max_ followed a clinal pattern. As latitude increases, T_br_ also increases, whereas T_opt_ and µ_max_ decrease (Table 2 and Fig. 3B and 3C). These indices showed a positive relation to annual maximum and minimum environmental temperatures (mean and extreme) but were not significantly correlated among them. Comparison of maximum rollover speed (µ_max_) showed significant differences among populations (ANOVA, F_2,27_ = 22.08; P<0.001), which are explained by higher values of µ_max_ in La Serena (Table 2; Fig. 3B; Tukey’s HSD tests, P<0.001). Similarly, significantly differences in the thermal optima across the geographical gradient (ANOVA, F_2,27_ = 9.14; P<0.01) are the result of contrasting scenarios with higher T_opt_ in snails from the lowest-latitude population and lower T_opt_ in snails from highest latitude population (Table 2; Fig. 3B; Tukey’s HSD tests, P<0.001).

### Recovery Time from Chill-coma

**Figure 4:**
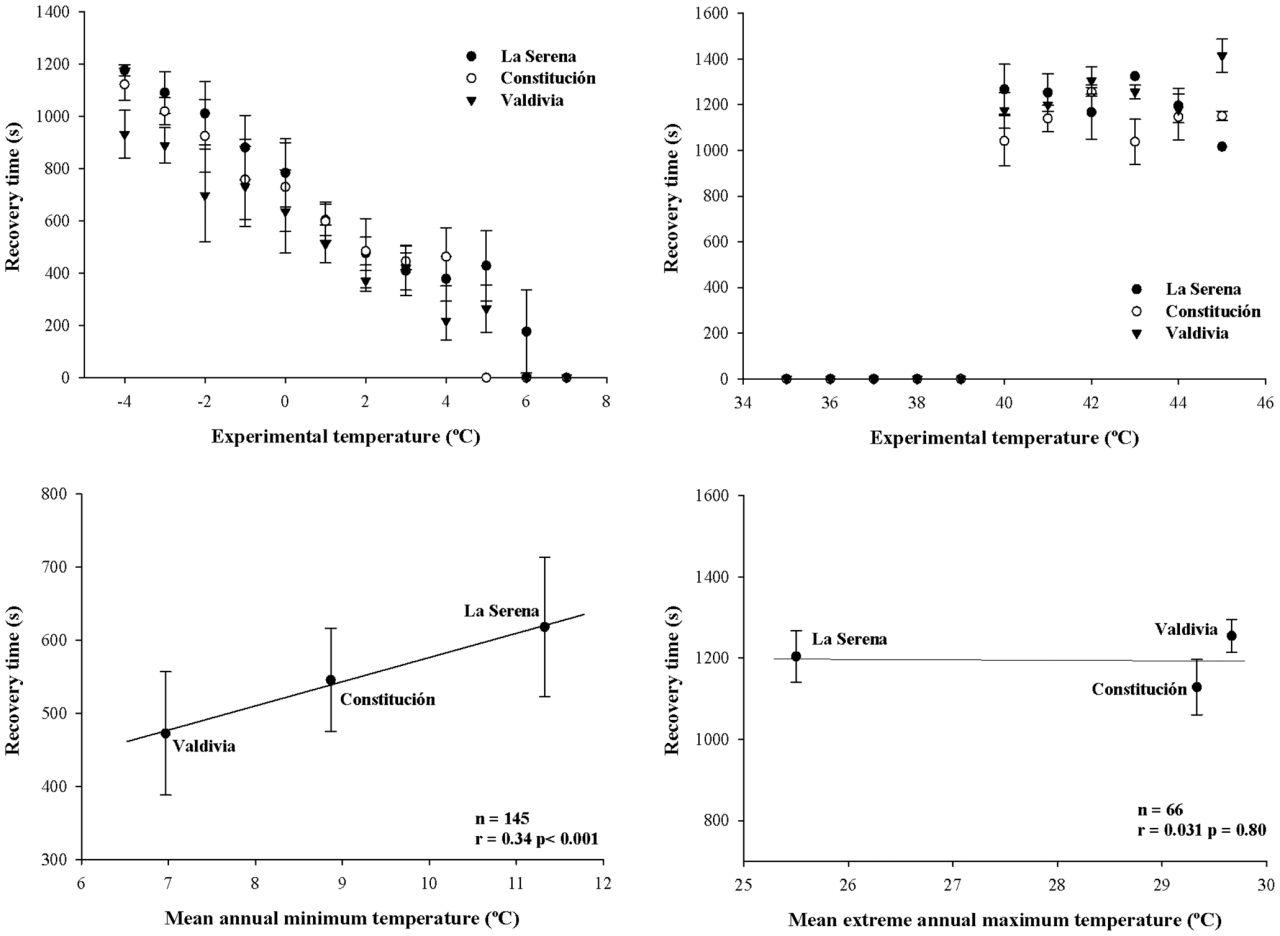
Geographic variation in recovery times from thermal coma. Relationship between experimental temperatures and population means of recovery time from (A) chill- and (B) heat-coma. Latitudinal variations in pooled means of recovery time from (C) chill-coma with the mean annual minimum temperature, and (D) heat-coma with the mean extreme annual maximum temperature. Lines show the trend between populations, circles and bars represent means ± SEM respectively.

**Figure 5:**
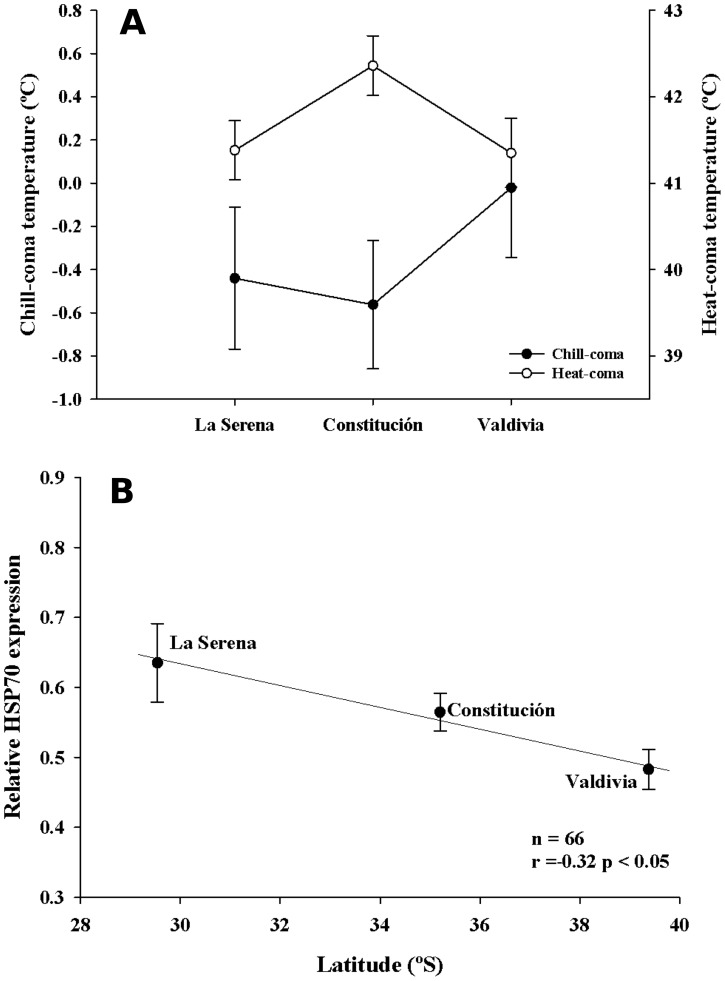
Geographic variation in thermal tolerances of *C. aspersum.* (A) Temperatures of chill- and heat-coma of the three populations of *C. aspersum*. (B) Latitudinal variation in the relative HSP70 protein expression in foot tissue of snail populations. Values are presented as means ± SEM. There were no differences detected between populations and thermal-coma temperatures (Xi^2^ test, P>0.05). Relative expression of HSP70 revealed differences between populations (ANOVA, P<0.05).

No significant correlations were found for recovery time or any other morphological and physiological measured trait. Overall, similar patterns of recovery time from chill-coma were found in the three populations of *C. aspersum* (Fig. 4A), with lower values at higher experimental temperatures (Fig. 4A). Between 7°C and 6°C, most of the snails did not enter a chill-coma, which was gradually manifested between 5°C and 2°C, and sharply visualized at these temperatures. Comparisons between populations and temperatures at which snails were in chill-coma revealed no differences (Fig. 4A, 5A; Xi^2^ = 9.69, df = 18, P = 0.93). However, analysis of recovery time between populations showed differences in this trait (Table 3), most of them explained by the lower values found in snails from Valdivia compared to the other two populations (Fig. 4C, Tukey’s HSD tests, P<0.05). Overall, recovery time followed the latitudinal distribution of the populations, showing a significant correlation with the mean annual minimum temperature (Fig. 4C). Snails from Valdivia (i.e., high latitude) exhibited the shortest recovery time of all populations, followed by Constitución and then, by La Serena (Fig. 4C). For those snails exposed to cold temperatures (i.e., from 7 °C to −4°C), there was no mortality detected, regardless of exposure time.

**Table 3:** Results for variance analyses (for body mass, chill- and heat-coma) and covariance (for standard metabolic rate and body dehydration) for the three populations of the land snail *C. aspersum.*

		MS	d.f.	*F*	*P*
**ANOVA**					
	**Body mass**				
	Population	0.021	2	2.48	0.09
	Error	0.001	311		
	**Chill-coma**				
	Population	672275	2	10.02	<0.001
	Error	67103	142		
	**Heat-coma**				
	Population	55987	2	1.16	0.32
	Error	48358	63		
	**HSP70**				
	Population	0.119	2	3.64	<0.05
	Error	0.033	63		
**ANCOVA**					
	**SMR (VCO_2Mean_)**				
	Population	0.009	2	0.40	0.67
	Error	0.021	310		
	**VCO_2Max_**				
	Population	0.016	2	1.24	0.29
	Error	0.013	310		
	**VCO_2Min_**				
	Population	0.005	2	0.39	0.67
	Error	0.012	310		
	**Body dehydration**				
	Population	3.91	2	11.16	<0.001
	Error	0.35	310		

MS = mean square; d.f. = degrees of freedom.

### Recovery Time from Heat-coma

The experimental heat treatment was similarly drastic for the three populations. All snails exposed to temperatures between 35°C and 39°C did not enter into a heat-coma (Fig. 4B), but as temperature increased from 39°C to 40°C, the recovery time increased sharply, and between 40°C to 45°C, mortality started to occur (some snails failed to recover after 2 hours). There were no differences detected between populations and temperatures at which snails were in heat-coma (Fig. 4B, 5A; Xi^2^ = 9.82, df = 10, P = 0.45). Additionally, no significant correlation was detected between recovery time from heat-coma and other morphological and physiological traits. Similarly, we did not find a correlation in recovery time with the mean maximum extreme environmental temperature in the latitudinal gradient (Fig. 4D), which was caused by the lack of differences in this trait between populations of *C. aspersum* (Table 3).

### Heat Shock Protein (HSP70) Expression

The HSP70 protein expression in foot tissue of the land snail *C. aspersum* did not show significant correlations with any physiological, morphological or performance measurement. Nevertheless, comparison of HSP70 relative expression after controlling with the constitutive β-actin protein, revealed differences between populations (Table 3), due to lower values in Valdivia compared to La Serena (Fig. 5B; Tukey’s HSD tests, P<0.05). Additionally, we found a significant correlation between the expression of the heat shock protein and latitude, showing that as the latitude increased, the HSP70 expression decreased (Fig. 5B).

### Standard Metabolic Rate

**Figure 6:**
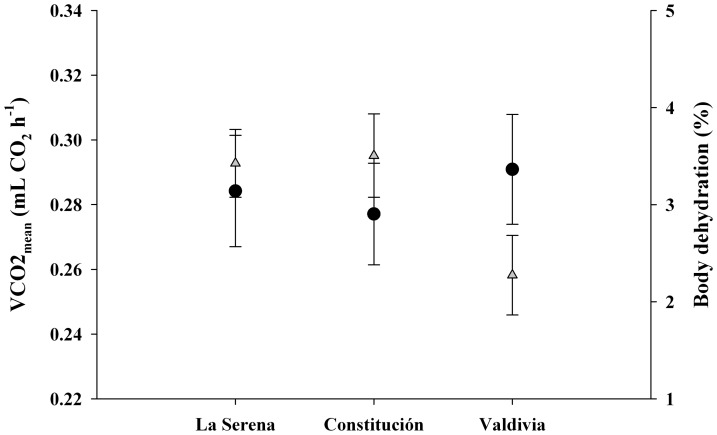
Geographic variation in physiological traits of the land snail *C. aspersum*. Standard Metabolic Rate (black circles) and Body Dehydration (gray triangles). Values are adjusted means ± S.E.M. No significant differences were found for Mb (ANOVA, P<0.05) or SMR (ANCOVA, P<0.05). BD showed significant differences after controlling for Mb (ANCOVA, P<0.05).

The mean values of physiological variables measured in the three populations of the land snail *C. aspersum* across a climatic gradient are summarized in Table 2. Body mass (M_b_) showed a positive correlation to standard metabolic rate (SMR, r = 0.60, P<0.05) and a negative correlation to BD (r = −0.22, P<0.05), but none of the metabolic traits were correlated to body dehydration (BD). Despite the lower values of M_b_ in La Serena (Table 2), no significant differences were found for this trait among populations (Table 3). After controlling for Mb, SMR did not show differences between populations (Fig. 6; Table 3), but BD showed significant differences (Fig. 6; Table 3), explained by a greater ability to maintain water balance (i.e., lower % body dehydration) in Valdivia compared to the other two populations (Fig. 3, Tukey’s HSD tests, P = 0.02).

## Discussion

### Geographical Variation in Thermal Sensitivities

Evolutionary theory of thermal biology predicts that any variation in the thermal sensitivities of snail populations as a result of thermal adaptation to their particular environmental conditions may manifest as shifts in the TPC parameters (i.e., CTs, µ_max_, T_opt_, and T_br_) [10], [14], [15], [59]. In our study, the shape and parameters of the TPCs revealed a latitudinal trend in snail thermal sensitivities, likely due to thermal adaptation across the geographical gradient. In fact, due to the great variety of environments in which *C. aspersum* inhabits in the world [40], [66]–[68], one could expect that its invasive success could be explained by a generalist thermal pattern (i.e., broadly adapted to a range of environments but not particularly well-adapted anywhere) [9], [16]. However, following our working hypotheses, we found evidence supporting the “hotter is better” scenario, since the populations that adapted to warmer temperatures (i.e., low latitude) showed higher optimal temperature and higher maximum performance (µ_max_) than the populations adapted to colder temperatures (i.e., high latitudes), demonstrating the existence of thermodynamic constraints. In spite of the aforementioned, the general picture of thermal sensitivity variation in the invasive snail *C. aspersum* is a bit more complex. We found evidence of a combination of “hotter is better” and “generalist-specialist trade-offs” hypotheses, supported by the differences in the thermal breadth (T_br_) between populations at the extremes of the geographical range, with La Serena population (i.e., warmer temperatures) exhibiting a narrower temperature range than Valdivia.

Some evolutionary explanations have been offered to account for these kinds of thermal patterns in natural populations. For instance, the combination of the “hotter is better” and the “generalist-specialist” patterns would emerge if lower µ_max_ evolved as a correlated response to selection for broader T_br_ in variable and unpredictable environments (e.g., high latitudes) [16], [69]–[73]. Thus, it is expected to find a trade-off between the T_br_ and µ_max_ as a result of directional selection for the evolution of broad thermal performance curves [26], [74]. This prediction is consistent with our data in which T_br_ and µ_max_ of snail populations showed opposite trends across the latitudinal gradient. Similar results have been documented in other organisms. For example, the temperature reaction norms of natural clones of the microbial eukaryote *Paramecium caudatum* evidenced signals of thermal generalism in populations at high latitudes (i.e., thermally variable environments), and signals of “hotter is better” in tropical populations as a result of local temperature adaptation [75]. However, it seems that such local thermal adaptation in *C. aspersum* populations is not totally achieved, because the lack of differences among populations in the upper and lower bounds for critical temperatures, as well as the absence of correlation between these thermal parameters with the local environmental temperatures (mean and extremes) [59]. The similar bounds for CTs across the latitudinal gradient could be the result of acclimation under common garden conditions [76], or some physiological and genetic constraints related to the recent introduction of this invasive snail in Chile [40]. In this sense, it has been recognized that differences in CTs across geographical gradients may not occur in populations that have previously lost genetic variability with respect to temperature sensitivity and tolerance due to their evolutionary history in a relatively uniform thermal environment [77]. This could be the case of *C. aspersum* snails in those populations that are considered the source of the Chilean populations (i.e., California coast and Mediterranean region) [40].

### Phenotypic and Molecular Variation in Thermal Tolerances

Some authors have argued that terrestrial ectotherms at higher latitudes require broader tolerance ranges (i.e., greater physiological plasticity) than individuals inhabiting in lower latitudes, as a way to deal with the greater thermal fluctuation that they experience [29], [78], [79]. In fact, ectothermic species differ widely in thermal tolerance limits and in their abilities to adjust these limits in temperature-adaptive manners [80], [81]. The thermal tolerances analyzed in our work revealed similar patterns in the recovery time from chill- and heat-coma among populations, with no differences in the temperatures at which snails enter into a thermal coma. Regardless of the aforementioned, snails from high latitudes (with colder and more variable temperatures) had faster recovery times from chill comas. The greater cold tolerance of high-latitude populations has been previously shown in other ectothermic species [64], [82]–[85], arguing that this thermal capacity likely enhances survival over winters [79], [85]. On the other hand, the experimental heat treatment was equally drastic for the three populations. Injuries produced by heat stress could be magnified at increasingly high temperatures, which were evidenced by the inability of some snails to recover from the heat-coma and death. In fact, the negative effects of high temperatures on the biological responses of ectotherms have been mainly associated with an oxygen limitation mechanism of thermal tolerance, together with the thermal sensitivity of macromolecular structures [38], [86], [87]. This may explain the lack of differences in the recovery time from the heat-coma and the absence of a latitudinal pattern in this thermal tolerance index among populations of *C. aspersum*. It has been shown that upper thermal limits and tolerances in terrestrial ectotherms, usually vary little across latitudinal gradients, while lower thermal limits and cold tolerance exhibit great variation at the intra- and inter-specific level [81].

The lack of differences in phenotypic responses to heat stress could be also explained, at least in part, by molecular compensations and/or adaptations (e.g., differences in HSP70 expression) to greater thermal variation. Indeed, heat shock protein response (HSPR) showed a clear sign of thermal compensation at the molecular level among populations in the geographical gradient. The relative expression of the HSP70 decreases as latitude increases, highlighting the possibility that those populations at higher latitudes require less molecular compensation to stabilize denaturing proteins, likely as a result of thermal adaptation (i.e., greater tolerance to thermal stress) to greater variation in environmental temperatures [36]–[39]. The rationale that explains these results is that the synthesis, degradation and replacement of these proteins imply an increase in energy costs for ectothermic organisms [88], [89].

### Physiological Differentiation across Latitude

It could be expected that the greater variation in environmental temperatures experienced by snails at high latitude influences the phenotypic responses in the life functions of these organisms, evidenced as changes in the rates of physiological and biochemical processes [3]–[5]. However, we found that, despite differences in environmental conditions exhibited by snail populations across the geographic gradient, their energy cost of maintenance (i.e., SMR) did not differ among populations. This is likely a result of (i) metabolic adjustments through phenotypic plasticity [29]; (ii) the minimization of climatic differences through a “microhabitat effect”, considered to be a behavioral adaptation to environmental heterogeneity affecting body temperature (e.g., orientation of the shell to facilitate heat flow or heat retention, climbing onto host plants to choose resting sites that are sheltered from harsh environmental conditions) [90]; (iii) the effect of common garden conditions and acclimation in laboratory [91]; or (iv) other compensatory shifts such as a greater ability to maintain water balance (i.e., lower % body dehydration) at higher latitudes than at lower latitudes. Other studies have shown that this physiological trait could contribute to variation in life history traits and fitness across a range of environments in other invasive molluscs [53], [92]. Therefore, this could be the result of physiological adaptation to greater variation in environmental temperatures during the year, which stimulates longer periods of dormancy (hibernation and aestivation) in the snails, as well as reduction of water loss [93].

Our results have shown that the invasive snail *C. aspersum* displays great variation in its thermal sensitivities and tolerances across geographic gradients. The thermal biology of this snail underlies its capacity to inhabit and colonize a wide range of climates, which may be favored by increased temperatures associated with climate change [94]. However, because temperature variability increases with latitude at a greater rate in the Northern versus the Southern Hemisphere [78], [79], then it is difficult to predict a future global scenario for this invasive species based only in our findings. One clue to this puzzle is found in the comparative study of Sunday et al. (2010) about the global variation of thermal tolerances in ectotherms across latitudinal gradients [81]. These authors found that thermal tolerance breadths generally increase with latitude, which is consistent with our results, and do so at a greater rate in the Northern Hemisphere [81]. Therefore, one would expect that thermal tolerances and capacities to respond to climate change in populations of *C. aspersum* at high latitudes in the Northern Hemisphere would be greater than those of the high latitude populations in the Southern Hemisphere.

### Conclusions

According to mtDNA analyses, the South American invasion of terrestrial snails from Europe is relatively recent (c.a. 100–150 years), and likely occurred through several introductions that are most probably on-going [40]. Hence, local differentiation in physiological traits is probably occurring, and is being buffered by gene flow among populations. The main conclusion that our study suggests is that physiological differentiation in Chilean *C. aspersum* snails is just emerging. Some traits (e.g., the ability to maintain water balance, TPCs parameters such as T_br_, T_opt_ and µ_max_, heat-shock protein expression, as well as recovery time from chill-coma) show differentiation, whereas others (CTs, standard metabolic rate, recovery time from heat-coma) do not. As a consequence, substantial variability in many traits exists among the analyzed snail populations, which – through the use of invasive species as model organisms – may reflect replicated experiments of thermal physiology evolution.
